# Classification of postoperative edema based on the anatomic division with mandibular third molar extraction

**DOI:** 10.1186/s40902-021-00291-w

**Published:** 2021-01-19

**Authors:** Yeong Kon Jeong, Jeong-Kui Ku, Sung Hyun Baik, Jae-Seek You, Dae Ho Leem, Sun-Kyu Choi

**Affiliations:** 1 Department of Oral and Maxillofacial Surgery, Section of Dentistry, Armed Forces Capital Hospital, Armed Forces Medical Command, Saemaul-ro 117, Bundang-gu, Seongnam-si, 13634 Republic of Korea; 2 Department of Radiology, Seoul National University Bundang Hospital, Seongnam, Republic of Korea; 3 Department of Oral and Maxillofacial Surgery, School of Dentistry, Chosun University, Gwangju, Republic of Korea; 4 Department of Oral and Maxillofacial Surgery, School of Dentistry and Institute of Oral Bioscience, Research Institute of Clinical Medicine of Jeonbuk National University-Biomedical Research Institute of Jeonbuk National University Hospital, Jeonbuk National University, Jeonju, Republic of Korea; 5 Department of Biostatistics, Korea University College of Medicine, Seoul, Republic of Korea

**Keywords:** Edema, Magnetic resonance imaging, Postoperative complications, Third molar, Tooth extraction

## Abstract

**Purpose:**

Several investigations have been performed for a postoperative edema after extraction, but the results have been controversial due to low objectivity or poorly reproducible assessments of the edema. The aim of this study was to suggest a classification and patterns of postoperative edema according to the anatomical division associated with extraction of mandibular third molar as a qualitative evaluation method.

**Methods:**

This study was conducted forty-four mandibular third molars extracted and MRI was taken within 48 h after extraction. The postoperative edema space was classified by MRI (one anatomic component—buccinator muscle—and four fascial spaces—supra-periosteum space, buccal space, parapharyngeal space, and lingual space), and evaluated independently by two examiners. The inter-examiner reliability was calculated using Kappa statistics.

**Results:**

The evaluation of buccinator muscle edema showed good agreement and the fascial spaces showed constant high agreement. The incidence of postoperative edema was high in the following order: supra-periosteum space (75.00%), buccinator muscle (68.18%), parapharyngeal space (54.55%), buccal space (40.91%), and lingual space (25.00%).

**Conclusion:**

Postoperative edema could be assessed clearly by each space, which showed a different tendency between the anatomic and fascial spaces.

## Background

Edema is the swelling of a part of the body due to fluid buildup in the tissues and is one of the major discomforts for patients after the extraction of a third molar with pain and trismus [1]. Inflammatory mediators are released after the surgical extraction, and an increase in vascular dilatation and permeability results in postoperative edema. Several studies have been conducted to evaluate and reduce the postoperative edema using modified surgical techniques, postoperative medication, and physiotherapy [2–4]. The postoperative edema gradually reaches the maximum by 48 h [3] and regresses by the fourth day with resolution 7 days after extraction [4]. The most common methods for evaluating postoperative edema are the subjective scale and objective craniometrics, measuring the overall facial swelling, using a range of indicators, such as flexible tape, drawing, or silk [5].

The conventional objective evaluation methods, on the other hands, have limitations in that the assessment is only for overall edema with low reproducibility of the measurement [6]. Several methods were reported to overcome these limitations [5, 7]; however, magnetic resonance imaging (MRI) is still the most objective assessment for the extent and area of swelling. Despite this, few studies have used MRI to measure swelling after a third molar extraction because of the cost and limited utility. Therefore, it is necessary to suggest new parameters for a postoperative swelling evaluation beyond the measurement of the swelling on MRI.

The military hospital has unique characteristics due to the nature of the lack of medical accessibility for military patients and can provide the medical service to the soldiers for free. With regard to the high risk of complications such as nerve damage after the extraction, the most accurate method, including MRI, can be used immediately after the extraction in the military hospital. Accordingly, we tried to develop an edema measurement method that can be used even in the absence of an MRI, using our MRI information from military hospitals.

An impacted mandibular third molar is adjacent to several anatomical structures that the postoperative swelling could spread. Two anatomical components (periosteum and buccinator muscle) and four fascial spaces (buccal, parapharyngeal, sublingual, and submandibular space) were considered to be clinically meaningful among the anatomic spaces around an impacted third molar. The aim of this study is to classify postoperative edema with MRI according to the anatomical division and to suggest patterns of the edema according to the anatomical spaces associated with the extraction of an impacted mandibular third molar.

## Materials and methods

**Fig. 1:**
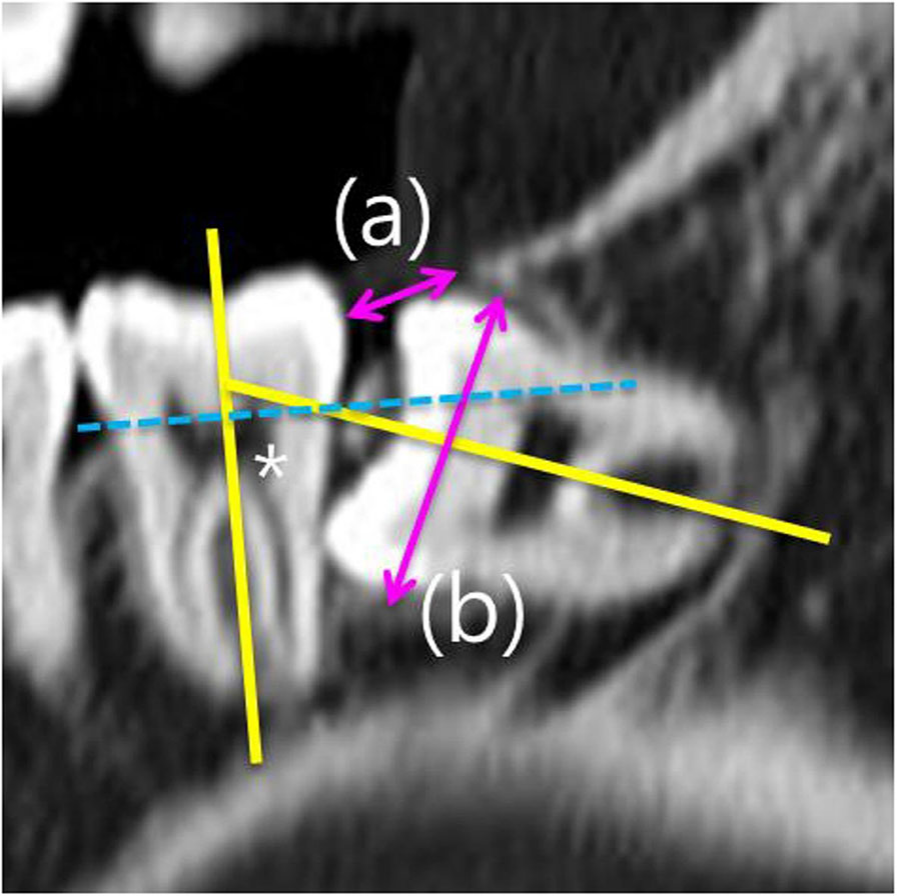
Assessment of the impacted third molar position in computed tomography according to a previous study [8]. The spatial relationship was classified based on the angle measured between the long axis of the impacted third molar and the adjacent second molar (yellow lines and an asterisk mark). Depth was classified based on the line connecting the cementoenamel junction of the adjacent second molar (dotted blue line)

This retrospective study included adult patients, who visited for the mandibular third molar extraction on the Department of Oral and Maxillofacial Surgery of Armed Forces Capital Hospital from May 2018 to February 2019. The inclusion criteria of the patients were as follows: (1) healthy status without any underlying disease, (2) impacted mandibular third molar which is horizontal (80 to 100°) with less than half of the third molar crown above the CEJ of the adjacent second molar and contacted with the inferior mandibular canal on computed tomography (Fig. 1) [8, 9], and (3) facial MRI within 48 h after surgical extraction of their mandibular third molar. The exclusion criteria were as follows: (1) simultaneous extraction of impacted maxillary third molar with incision, ostectomy, or odontomy, (2) poor oral hygiene control, and (3) uncontrolled systemic diseases.

Surgical extraction of the mandibular third molar was performed with operculectomy. After operculectomy, the extraction was performed through odontomy on the exposed third molar with or without additional mesial incision [8]. Patients were instructed to take oral antibiotics (625 mg, amoxicillin, Ilsung Pharmaceutical, Korea) and NSAID (500 mg, dexibuprofen, Samil Pharmaceutical, Korea) thrice daily for 5 days and daily mouth rinse with a chlorhexidine solution.

In this study, the facial MRI (Discovery™ MR750, GE Healthcare, USA) was taken within 48 h after the extraction. The MRI protocol included T2-weighted nonfat-saturated fast spin-echo sequence (TR, 2800 ms; TE, 90 ms; matrix, 320 × 224; slice thickness, 5 mm; gap, 1 mm; FOV, 34 × 34 mm^2^) and T1-weighted-echo sequence with fat suppression (TR, 3.5 ms; TE, 1.6 ms; matrix, 288 × 160; FOV, 40 × 28 mm^2^; section thickness, 5 mm). The postoperative edema space was determined by the high signal in the T2-weighted MRI image and the low signal in the T1-weighted MRI and was evaluated independently by one expert oral and maxillofacial surgeon (Y.K.J) and radiologist (B.S.H).

Ethical approval was approved by the Institutional Review Board at Armed Forces Capital Hospital (No. AFCH-19-IRB-008) and followed the STROBE Guidelines with the Helsinki Declaration.

**Fig. 2:**
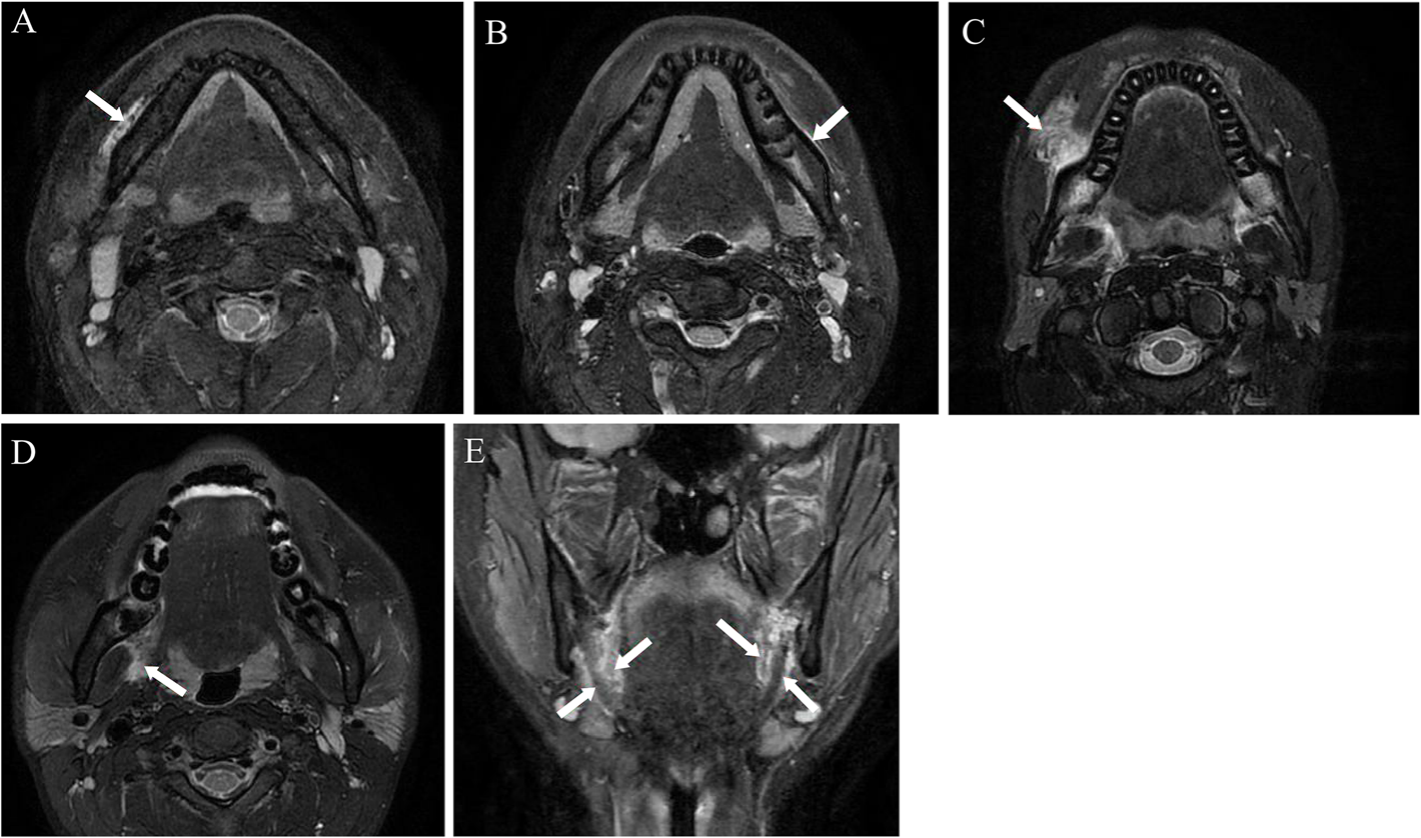
Examples of edema in the postoperative spaces on magnetic resonance imaging. **a** Buccinator muscle (arrow). **b** Supra-periosteum space (edema space observed along the outer border of the mandible, arrow). **c** Buccal space (arrow). **d** Parapharyngeal space (arrow). **e** Lingual space (edema space on sublingual or submandibular space, arrows)

In MRI, the spaces around the mandibular third molar were divided into the buccinator muscle, supra-periosteum space, buccal space, parapharyngeal space, and lingual space (sublingual and submandibular space) [10, 11]. Each space was defined as follows:

1. *Buccinator muscle* (Fig. 2a). An anatomical component organized from the origin (from the alveolar processes of the maxilla and mandible, buccinators crest, and temporomandibular joint) to insertion (in the fibers of the orbicularis oris muscle).
2. *Supra-periosteum space* (Fig. 2b). When enhancement is observed along the outer border of the mandible on MRI, it corresponds anatomically to the periosteum, but it is defined as the supra-periosteum space because the periosteum cannot be stretched enough to allow fluid collection and is attached firmly to the underlying bone.
3. *Buccal space* (Fig. 2c). The fascial space with a buccal fat pad consists of anterior (angle of the mouth), posterior (masseter muscle), superior (zygomatic process of the maxilla and zygomaticus muscles), inferior (depressor anguli oris muscle and attachment of the deep fascia to the mandible), medial (buccinator muscle), and lateral (platysma muscle, subcutaneous tissue, and skin) borders.
4. *Parapharyngeal space* (Fig. 2d). The fascial space consists of anterior (pterygomandibular raphe), posterior (deep lobe of the parotid gland), superior (lateral pterygoid muscle), inferior (attachment of medial pterygoid of the mandible), medial (medial pterygoid muscle), and lateral (medial surface of ramus of the mandible) borders.
5. *Lingual space* (sublingual and submandibular space, Fig. 2e). The fascial space on the lingual side of mandible consists of anterio-lateral (medial surface of the mandible), superior (mucosa of the floor of mouth and the tongue), posterior (hyoid bone), and inferio-lateral (platysma muscle and superficial layer of the deep cervical fascia) borders.

### Statistical analysis

The inter-examiner reliability was calculated using Kappa statistics. Kappa values were rated as follows: < 0.200 was considered poor, 0.201–0.400 fair, 0.401–0.600 moderate, 0.601–0.800 good, and > 0.800 excellent [12]. Except for the inconsistent postoperative images of edema between the two examiners, the correlation of the postoperative incidence of edema among each space was analyzed using a Pearson Chi-square test. In addition, the differences between the patients who underwent extraction only mandibular third molar or with maxillary third molar, and under local or general anesthesia were analyzed using a Fisher exact test and Chi-square test. Two-sided *P* values of < 0.05 were considered significant. The analysis was performed using SPSS 25.0 for Windows (SPSS Inc., Chicago, IL, USA). The continuous variables were expressed as mean ± standard deviation (SD) and qualitative variables as absolute and percent frequencies.

## Results

**Fig. 3:**
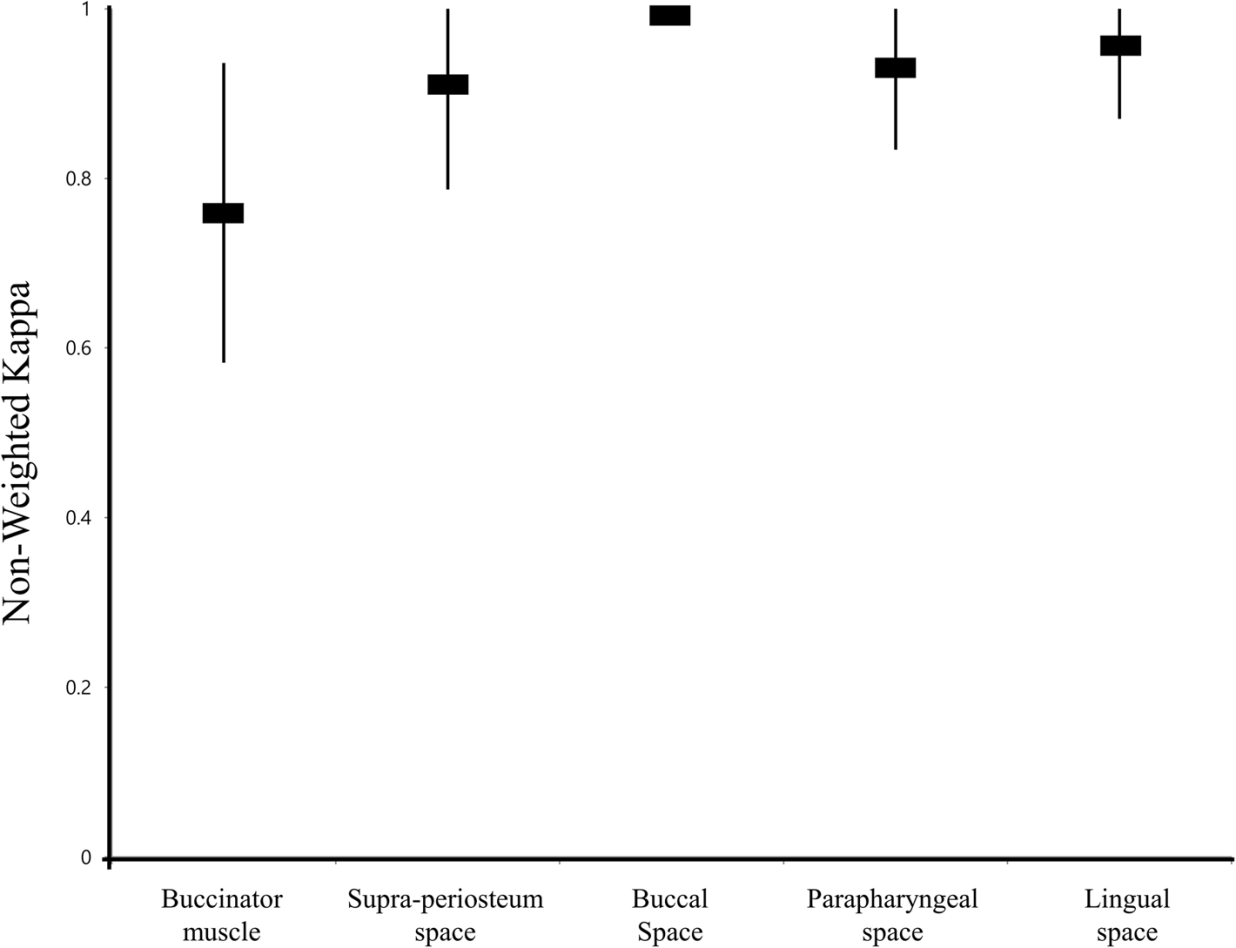
Kappa agreement between the two examiners for each postoperative edema space

MRI was taken after the extraction of 57 mandibular third molars from 48 male patients (20.3 ± 0.58 years) (Table 1). The evaluation of the buccinator muscle swelling showed good agreement (Kappa, 0.716; 95% CI, 0.528–0.905). On the other hand, there was no disagreement when considering the swelling on the buccal space (Kappa, 1.000). The supra-periosteum space (Kappa, 0.910; CI, 0.787–1.000), parapharyngeal space (Kappa, 0.929; CI, 0.834–1.000), and lingual space (Kappa, 0.956; CI, 0.870–1.000) showed excellent agreement (Fig. 3). Overall, high mean Kappa values were calculated, and the inter-examiner agreement was constant among the spaces.

**Table 1:** Demographic and clinical information of the patients

Number of patients	48
Sex	All male
Age	20.3 ± 0.58 years
Anesthetic method (local:general)	24:20
Simultaneously extraction of maxillary third molar (only mandibulular third molar:with maxillary third molar)	9:35

**Fig. 4:**
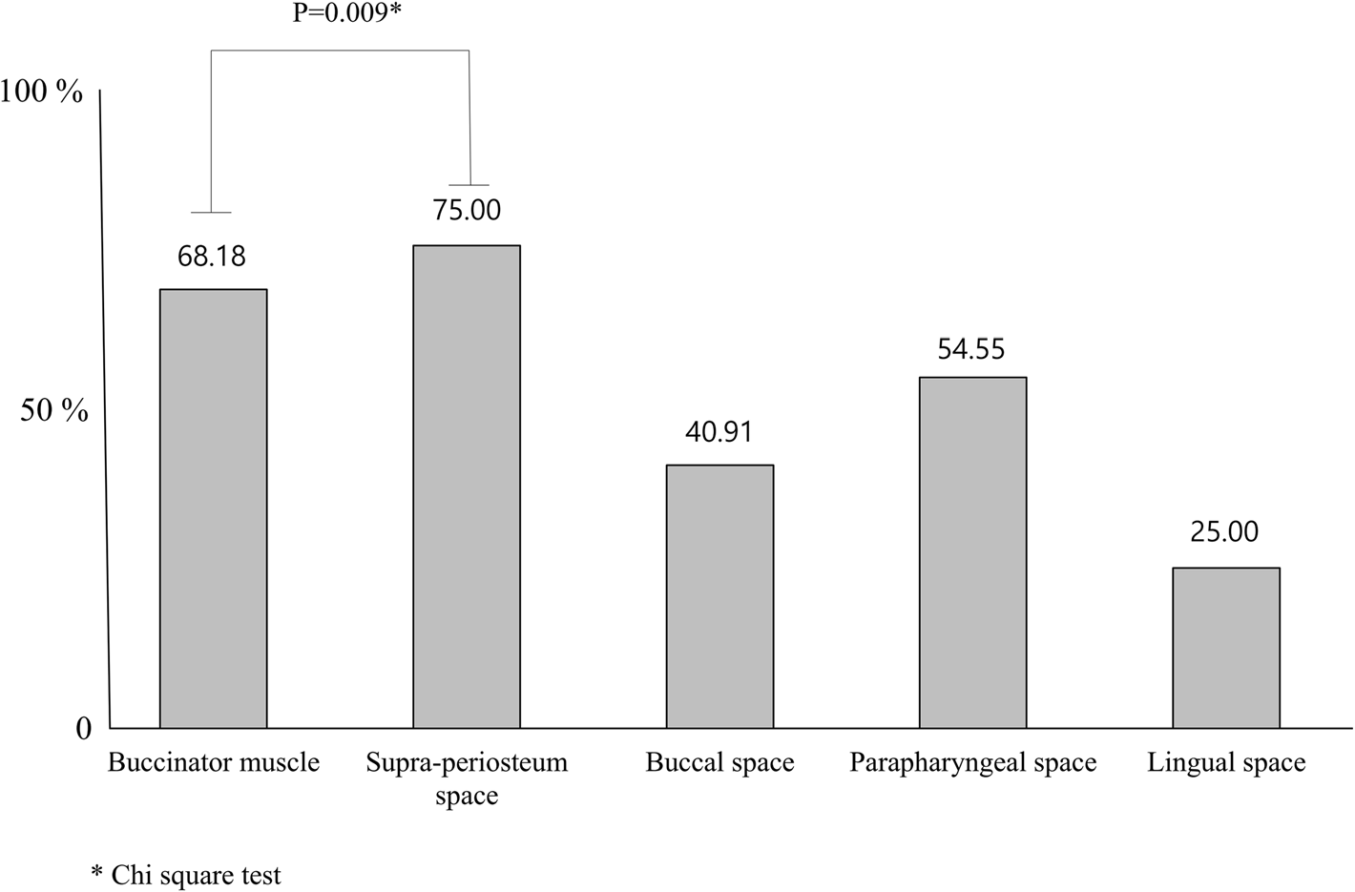
Incidence of postoperative edema for each space. The incidence of buccinator muscle and supra-periosteum space showed a correlation according to a Chi-square test (*P* = 0.028)

Except for the nine extractions with inconsistent MRI images, 44 extractions were analyzed for the postoperative edema space tendency according to the variables. Overall, the incidence of postoperative edema was 75.00% on the supra-periosteum space, 68.18% on the buccinator muscle, 40.91% on the buccal space, 54.55% on the parapharyngeal space, and 25.00% on the lingual space. Among the spaces, the incidence of edema in the buccinator muscle and supra-periosteum space showed a significant correlation (*P* = 0.009, Fig. 4).

Twenty-four and 20 of the teeth were extracted under local and general anesthesia, respectively. Extractions under general anesthesia showed frequent edema in the buccinator muscle (79.17 vs. 62.96%, *P* = 0.519), supra-periosteum space (78.57 vs. 70.37%, *P* = 0.728), buccal space (57.14 vs. 37.93%, *P* = 0.760), and lingual space (32.14 vs. 21.43%, *P* > 0.999), but not in the parapharyngeal space (46.15 vs. 62.07%, *P* > 0.999); however, the differences were not statistically significant (Table 2).

**Table 2:** Incidence of postoperative edema for each space according to the anesthetic methods and maxillary third molar extraction

	*N*	Time to MRI (h, SD)	Postoperative edema (%)				
			Buccinator muscle	Supra-periosteum space	Buccal space	Parapharyngeal space	Lingual space
Anesthetic method							
Local	24	4.71 (9.41)	62.96	70.37	37.93	62.07	21.43
General	20	17.65 (20.98)	79.17	78.57	57.14	46.15	32.14
P*			0.519	0.728	0.760	> 0.999	> 0.999
Simultaneous extraction of maxillary third molar							
Only mandibular third molar	9	7.56 (15.22)	60.00	63.64	41.67	41.67	0.00
With maxillary third molar	35	11.37 (17.38)	73.17	77.27	48.89	58.14	34.09
P*			0.298	0.669	> 0.999	0.057	0.085

*A *P* value of < 0.05 was considered statistically significant with Fisher exact test

Thirty-five and 9 extractions were performed simultaneously with the maxillary third molar and only the mandibular third molar, respectively. The extractions with the maxillary third molar showed frequent edema than that of the mandibular third molar extraction only on all spaces without statistical significance: buccinator muscle (73.17 vs. 60.00%, *P* = 0.298), supra-periosteum space (77.27 vs. 63.64%, *P* = 0.669), buccal space (48.89 vs. 41.67%, *P* > 0.999), parapharyngeal space (58.14 vs. 41.67%, *P* = 0.057), and lingual space (34.09 vs. 0.00%, *P* = 0.085) (Table 2).

## Discussion

The authors hypothesized that postoperative edema related with the surgical extraction of impacted mandibular third molar could be separated by anatomic divisions. The periosteum, as a highly vascularized tissue, could play a role in edema drainage [13]. In this study, edema was observed frequently along the outer border of the mandible. Radiologically, this condition is barely regarded as edema of the periosteum because it is a thin structure and attaches firmly to the underlying bone. Nevertheless, there were also no other anatomical structures known to exist along the mandibular surface. The authors assumed that it was either a loose connective tissue space for muscular fiber insertion into the periosteum or an unknown fascial space, and it was defined as the supra-periosteum space. As a result, the five postoperative edema spaces (one anatomic structure—buccinator muscle—and four fascial spaces—supra-periosteum, buccal, parapharyngeal, and lingual space) were classified clearly with substantial reliability.

The frequency of postoperative edema was observed in the order of the supra-periosteum space; buccinator muscle; and parapharyngeal, buccal, and lingual spaces. A significant correlation was observed between the incidence of edema on the supra-periosteum space and the buccinator muscle (*P* = 0.009). Therefore, most postoperative edema could anteriorly spread to the supra-periosteum space and buccinators muscle. In contrast to the muscle fibers, which are inserted into the bone and have natural circulation to the bone, the fascial spaces do not have connected anatomic structures with the bone [10]. Without the invasion of the anatomic structure during the operation, postoperative edema may have a difficulty to spread toward the fascial space. Considering the extraction process, a distal (ramus area) incision, with the invasion of pterygomandibular raphe, might be related to edema in the parapharyngeal space. The damage to the lingual plate might be related to lingual space edema.

With regard to reduced swelling after surgical extraction, the basis could be established by the pattern of postoperative edema in each space. The clinical swelling could be associated with edema of the buccinator muscle, buccal, and supra-periosteum spaces, which are located in the buccal side of the mandible. The parapharyngeal and sublingual spaces could be associated with neck and swallowing discomfort. Considering this edema pattern, the authors noted the possibility of anatomic discontinuity on the buccinator muscle or periosteum to allow edema to spread into the buccal space. The buccal space is one of the fascial spaces and its volume can be extended easily to cause facial swelling and drain slowly via the cutaneous sinus at the inferior of the space [14]. In accordance with some studies, this possibility was supported by their results of significant swelling in the flap design with a mesial vertical incision [15–17]. In addition, this possibility supported a clinical study of flap design for guided bone regeneration in that flap management without a vertical incision can avoid cutting the muscle fibers, leading to a decrease in postoperative swelling and wound dehiscence [18]. Therefore, an incision without invasion of the anatomic structures, such as the buccinator muscle and periosteum, can reduce the level of postoperative edema.

Many studies have been conducted on the contributing factors, including incision design and affected postoperative swelling [2]. However, previous research on postoperative swelling has been controversial because the clinical methods used to evaluate swelling (such as a questionnaire or distance measurement between facial reference points) were not objective and reproducible [5, 6]. Therefore, the authors suggest region-specific edema assessment of mandibular third molar extraction as a qualitative method. The postoperative edema in each space could be classified independently, leading interpretation of various postoperative edema degrees and allowing the symptoms to be estimated according to each space. In the limitation of this retrospective study, the authors could not correlate the radiological (MRI) edema with surgical procedure and clinical symptoms. Further clinical studies should be necessary to reveal and reduce postoperative edema by considering each divided space.

## Conclusion

An evaluating postoperative edema based on anatomic division showed high reliability according to the space classification, which was divided into five spaces: one anatomic structure (buccinator muscle) and four fascial spaces (supra-periosteum, buccal, parapharyngeal space, and lingual space). Most postoperative edema was spread though buccinators muscle and supra-periosteum space with a significant correlation. Further study should be conducted for the clinical validation of this classification according to the relevance with the swelling.

## Data Availability

The datasets used during the current study are available from the corresponding author on reasonable request.
